# Effects of Volatile Organic Compounds on Biofilms and Swimming Motility of *Agrobacterium tumefaciens*

**DOI:** 10.3390/microorganisms10081512

**Published:** 2022-07-26

**Authors:** Daria E. Sidorova, Mariia I. Skripka, Inessa A. Khmel, Olga A. Koksharova, Vladimir A. Plyuta

**Affiliations:** 1 Institute of Molecular Genetics of National Research Center “Kurchatov Institute”, Kurchatov Sq. 2, 123182 Moscow, Russia; misenok1@gmail.com (D.E.S.); mariya2010_14@mail.ru (M.I.S.); khmel@img.ras.ru (I.A.K.); koksharova@belozersky.msu.ru (O.A.K.); 2 Department of Biotechnology, Mendeleev University of Chemical Technology of Russia, 125480 Moscow, Russia; 3 A.N. Belozersky Institute of Physico-Chemical Biology, Lomonosov Moscow State University, Leninskie Gory 1-40, 119991 Moscow, Russia

**Keywords:** biofilms, volatile organic compounds, swimming motility, *Agrobacterium tumefaciens*, ketones, alcohols, terpenes

## Abstract

Volatile organic compounds (VOCs) emitted by bacteria play an important role in the interaction between microorganisms and other organisms. They can inhibit the growth of phytopathogenic microorganisms, modulate plant growth, and serve as infochemicals. Here, we investigated the effects of ketones, alcohols, and terpenes on the colony biofilms of plant pathogenic *Agrobacterium tumefaciens* strains and swimming motility, which can play an important role in the formation of biofilms. It was shown that 2-octanone had the greatest inhibitory effect on biofilm formation, acting in a small amount (38.7 g/m^3^). Ketone 2-butanone and unsaturated ketone β-ionone reduced the formation of biofilms at higher doses (145.2–580.6 and 387.1–1548.3 g/m^3^, respectively, up to 2.5–5 times). Isoamyl alcohol and 2-phenylethanol decreased the formation of biofilms at doses of 88.7 and 122.9 g/m^3^ by 1.7 and 5 times, respectively, with an increased effect at 177.4 and 245.9 g/m^3^, respectively. The agrobacteria cells in mature biofilms were more resistant to the action of ketones and alcohols. These VOCs also suppressed the swimming motility of agrobacteria; the radius of swimming zones decreased ~from 2 to 5 times. Terpenes (−)-limonene and (+)-α-pinene had no significant influence on the colony biofilms and swimming motility at the doses used. The results obtained represent new information about the effect of VOCs on biofilms and the motility of bacteria.

## 1. Introduction

In recent years, volatile organic compounds (VOCs) released by microorganisms have aroused great interest among researchers working in the field of microbiology, biotechnology, medicine, and agriculture. VOCs are lipophilic compounds with low molecular masses (on average, 300 Da), low boiling points, and high vapor pressure. VOCs can spread through air and liquids, acting over short and long distances [1,2,3,4,5,6,7,8]. Bacterial VOCs belong to various chemical types, including ketones, alcohols, terpenoids, sulfur-containing compounds, alkenes, etc. A database of identified VOCs (mVOC 3.0 database, https://bioinformatics.charite.de/mvoc/ accessed on 23 July 2022) has been published; it includes more than 2000 compounds emitted by about 1000 species of bacteria and fungi [9]. However, this is only a small part of volatile substances and their producers due to the complexity of their identification and the small number of studied strains of microorganisms.

It has been shown that VOCs emitted by microorganisms can play a significant role in interactions between microorganisms. VOCs can modulate the growth and development of microorganisms and plants (inhibit or stimulate); cause systemic resistance of plants; and affect insects, nematodes, and other organisms. The formation of VOCs can be important in microbial competition within a specific ecological niche (e.g., in the rhizosphere of plants, in soil), the antagonistic relations between plant-pathogenic and plant-associated bacteria, and microorganisms of the microbiota of humans and animals [1,6,10,11,12,13,14,15]. In addition, VOCs can play a significant role in a new type of communication between organisms and act as infochemicals capable of transmitting information over long distances [2,3,4,12,15].

Earlier, we found out that the ketones 2-nonanone, 2-undecanone, 2-heptanone, sulfur-containing compound dimethyl disulfide (DMDS), and alkene 1-undecene emitted by *Pseudomonas* and *Serratia* strains have an inhibitory and killing effect on the phytopathogenic bacteria *A. tumefaciens,* the insect *D. melanogaster*, the nematode *Caenorhabditis elegans* [16], and the plant *A. thaliana* [17].

Recently, we investigated the biological activity of eight pure VOCs of various types. We showed [18] that of the three volatile ketones studied, the most significant inhibition of *A. tumefaciens* growth was observed with the action of 2-octanone (at low doses of 25–100 µmol (32.26–129.04 g/m^3^)), and inhibitory actions of the higher amounts of 2-pentanone and 2-butanone were found. Isoamyl alcohol had an inhibitory effect at low doses (25–100 µmol (22.18–88.71 g/m^3^)) and another alcohol, 2-phenylethanol, had a weaker effect on *Agrobacterium* growth at doses from 25–100 µmol (30.74–122.94 g/m^3^). β-Ionone had a weak inhibitory effect on agrobacteria at high doses (200–800 µmol (387.07–1548.3 g/m^3^)). Two terpenes, (–)-limonene and (+)-α-pinene, practically did not inhibit the growth of agrobacteria even at a dose of 400 (548.46 g/m^3^) ((–)-limonene) and 600 μmol (822.63 g/m^3^) ((+)-α-pinene). The effects of these VOCs on the growth of *A. thaliana* and seed germination were also investigated. Interestingly, the volatile ketones 2-butanone and 2-pentanone increased the biomass of *A. thaliana* at doses of 200–400 μmol (145.15–290.3 and 173.4–346.73 g/m^3^, respectively) by 1.5–2 times; 2-octanone had the same effect at 10 μmol (12.9 g/m^3^), but with a further increase in its amount, the biomass of plants decreased. Isoamyl alcohol and 2-phenylethanol suppressed plant biomass several times at doses of 50–100 μmol (44.36–88.71 and 61.47–122.94 g/m^3^, respectively), and β-ionone and (–)-limonene had a noticeable inhibitory effect only at a high dose (600 μmol (1161.21 and 822.69 g/m^3^, respectively)). The germination of plant seeds was most strongly suppressed by isoamyl alcohol (50–100 μmol (44.36–88.71 g/m^3^)) and 2-phenylethanol (25–200 μmol (30.74–245.89 g/m^3^)). A substantial killing effect on *D. melanogaster* was exerted by terpenes and ketones (2-octanone and 2-pentanone). Thus, the sensitivity of organisms belonging to different kingdoms to VOCs was different [18].

In the natural environment, bacteria usually exist in the form of biofilms, multicellular communities enclosed in a matrix and attached to various surfaces. The ability of bacteria to exist in biofilms causes serious problems in medicine, biotechnology, and agriculture, since bacteria living inside biofilms have a significantly higher resistance to antibacterial agents, including antibiotics, pesticides, etc. Pathogenic bacteria can form biofilms on various implantable devices, which leads to intractable chronic infections [19,20]. Biofilms associated with plants and phytopathogenic bacteria in the rhizosphere play a significant role in the interactions of microorganisms with plants [21,22,23]. The ability of phytopathogenic bacteria, including *Agrobacterium* species, to form biofilms on plants is considered one of the virulence factors in bacterial infections.

In connection with the above, the search for and study of substances capable of suppressing the formation of biofilms are extremely important tasks. In this regard, it seems very promising to study the influence of various VOCs on the formation of biofilms and the survival of bacteria in them. This issue has been little studied. The huge amount of VOCs emitted by microorganisms opens up great opportunities for these studies.

Previously, we studied the effect of three volatile ketones (2-nonanone, 2-heptanone, 2-undecanone) and a sulfur compound (DMDS) on static colony biofilms formed by three strains of *A. tumefaciens* and showed the inhibitory action of these VOCs on the formation of biofilms of *A. tumefaciens* strains [24]. In this work, we investigated the effect of several other pure VOCs, belonging to three different chemical groups (ketones, alcohols, terpenoids) that can be released by various bacteria and fungi (mVOC 3.0 database [9]), on *A. tumefaciens* colony biofilms. In addition, the effect of VOCs on bacterial motility, which is important for biofilm formation, was also studied.

## 2. Materials and Methods

### 2.1. Bacteria and Conditions of Growth

The following strains of *A. tumefaciens* were used in this work: *A. tumefaciens* C58, isolated from cherry crown gall (USA), nopaline-type [25]; *A. tumefaciens* Chry5, isolated from *Chrysanthemum* crown gall (USA), chrysopine-type [26]. Bacteria were grown in liquid Luria–Bertani broth (LB) or on solid (1.5% *w*/*v* agar) Luria–Bertani agar (LA) (Sigma-Aldrich Chemie GmbH, Steinheim, Germany) at 28 °C.

**Figure 1:**
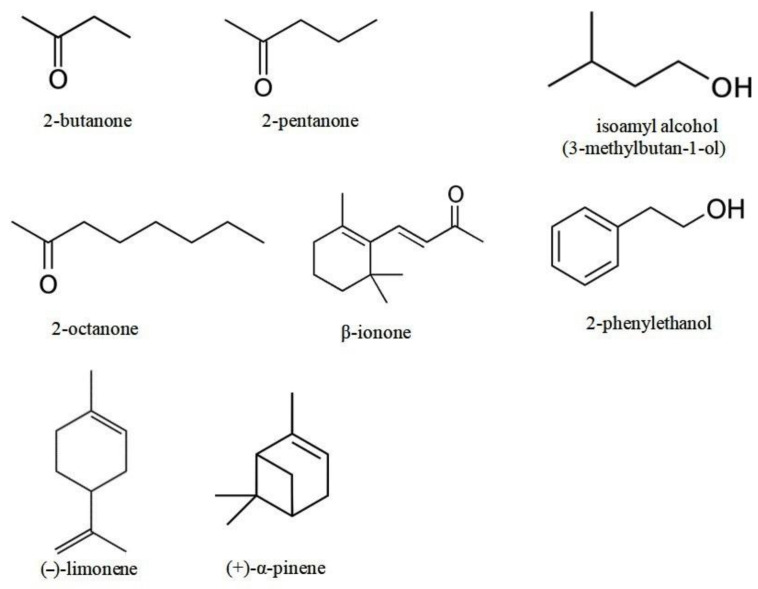
Volatile organic compounds used in this work.

The pure VOCs of the following classes were studied (Figure 1): alcohols (2-phenylethanol and isoamyl alcohol; purity of both is >99%); ketones (2-butanone, 2-pentanone, 2-octanone, unsaturated ketone, and norterpenoid β-ionone; all of them had >99% purity); and terpenes ((–)-limonene with purity > 96% and (+)-α-pinene with purity > 98%). All compounds were obtained from Sigma-Aldrich Chemie GmbH, Steinheim, Germany. VOCs were taken directly from the initial liquid preparation without dilution in a solvent.

### 2.2. Detection of the Effect of VOCs on the Formation of Colony Biofilms

The effect of the VOCs on biofilm formation was determined as described [24,27,28]. Isopore^TM^ track-etched polycarbonate membrane filters (25 mm in diameter; 0.2 µm pore size, Millipore, Sigma-Aldrich Chimie GmbH, Steinheim, Germany) were sterilized by UV exposure for 20 min for each side. Planktonic cultures of the tested *A. tumefaciens* strains grown in LB medium under shaking for 24 h at 28 °C were diluted with fresh LB medium to a value of 4 ± 1 × 10^6^ cells mL^−1^. Membrane filters were inoculated with 15 µL of diluted culture and placed on LA medium (7 mL) in one part of the bi-partitioned plastic Petri dishes (diameter 92 mm, height 16 mm, the headspace volume of the Petri dish is approximately 99.4 cm^3^). Chemical preparations of individual VOCs or sterile H_2_O (control) (15 µL) were placed on the sterile filter paper in another section of the Petri dish. The plates were tightly sealed with Parafilm M (Pechiney Plastic Packaging Company, Chicago, IL, USA) in 4 layers, and incubated at 28 °C for 48 h. Then, each membrane filter carrying biofilm was placed in 5 mL of 0.85% aqueous NaCl water solution, vigorously mixed, and the colony-forming units (CFUs) in the biofilm were determined by the plating of serial dilutions on the LA plates.

### 2.3. Detection of the Effect of VOCs on Bacterial Cell Survival in Mature Biofilms 

The effect of the VOCs on mature biofilms was determined as described [24,27,28]. The biofilms of *A. tumefaciens* strains were grown for 48 h on membrane filters on the LA plates sealed with Parafilm M. Then, the membrane filters coated with mature biofilms (defined by the time of their formation, 48 h) were replaced on the fresh LA medium on one side of the bi-partitioned Petri dish. Individual VOCs or 15 µL of sterile H_2_O (control) were placed on the sterile filter paper in another part of the plate. Then, the plates were tightly sealed with Parafilm M and incubated at 28 °C for an additional 24 h. After that, each membrane filter carrying a biofilm was placed in 5 mL of 0.85% NaCl sterile solution, mixed by vortexing, and the CFUs of *A. tumefaciens* cells were determined by the plating of serial dilutions on the LA plates.

### 2.4. Influence of VOCs on the Motility of A. tumefaciens Strains

Overnight cultures of the *Agrobacterium* strains were diluted 100 times in a fresh LB medium and grown for 2–3 h until cells reached the exponential growth phase. Swimming motility: 3 μL of bacterial culture was applied to the surface of the growth medium with 0.3–0.33% agar: LB or M9 with additives (0.4% glucose; 0.5% casamino acids) on one side of a two-section Petri dish. VOCs or sterile H_2_O (control) (15 µL) were placed on the sterile filter paper in another part of the plate. The plates were sealed carefully with Parafilm M and incubated for 36 h at 28 °C. The presence of zones of the swimming motility of bacteria on the surface of the medium was determined visually. The radius of the swimming zones was measured with a ruler. Twitching motility: cells were stab inoculated with a toothpick through a thin (approximately 3 mm) LB agar layer (1% agar) to the bottom of the Petri dish. After incubation for 24 to 48 h at 28 °C, hazy zones of growth at the interface between the agar and the surface of the Petri dish were observed [29]. Swarming motility was determined on M9 agarized (0.6% agar) medium containing 0.4% glucose and 0.5% casamino acids (Difco) or on LA (0.5% agar). In total, 10 μL of culture was applied to the surface of the media, and the plates were incubated at 28 °C [30].

The properties of VOCs, such as volatility (vapor pressure) and lipophilicity, studied amount, and concentration of VOCs, are presented in Table 1.

### 2.5. Statistical Analysis

Statistical analysis was performed using analysis software IBM SPSS software v. 26 (New York, NY, USA). The mean values and standard errors were calculated using the Excel descriptive statistics program for the on-plate assays. Significant differences were determined by one-way ANOVA followed by Tukey’s HSD (honestly significant difference) post hoc test. Differences were considered to be significant at *p* ≤ 0.05. All experiments were repeated three times with three plates for one dose of the VOC and three filters with biofilms per dish (a total of 9 replicates (*n* = 9) per independent replicate).

## 3. Results

### 3.1. The Effects of VOCs on A. tumefaciens Colony Biofilms

**Figure 2:**
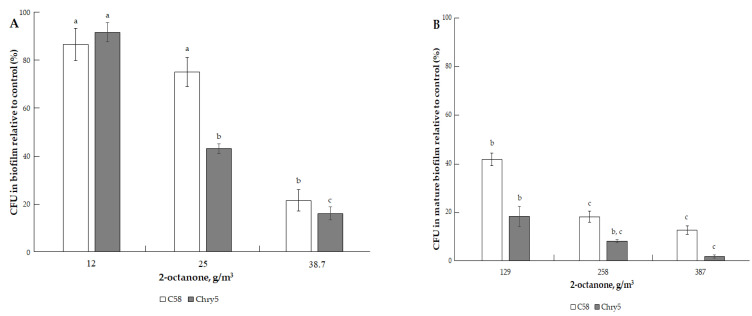
The effect of 2-octanone on the formation of biofilm of *A. tumefaciens* C58 (white column) and Chry5 (gray column) (**A**) and cell survival in mature biofilms (**B**) depending on the concentration of 2-octanone. CFU of bacteria strains in the control (without VOC): 100^a^%. The different lowercase letters above the mean values indicate significant differences (*p* ≤ 0.05; Tukey’s HSD test).

**Figure 3:**
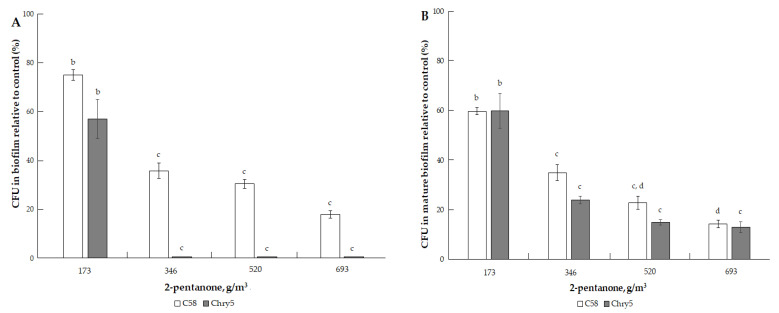
The effect of 2-pentanone on the formation of biofilm of *A. tumefaciens* C58 (white column) and Chry5 (gray column) (**A**) and cell survival in mature biofilms (**B**) depending on the concentration of 2-pentanone. CFU of bacteria strains in the control (without VOC): 100^a^%. The different lowercase letters above the mean values indicate significant differences (*p* ≤ 0.05; Tukey’s HSD test).

**Figure 4:**
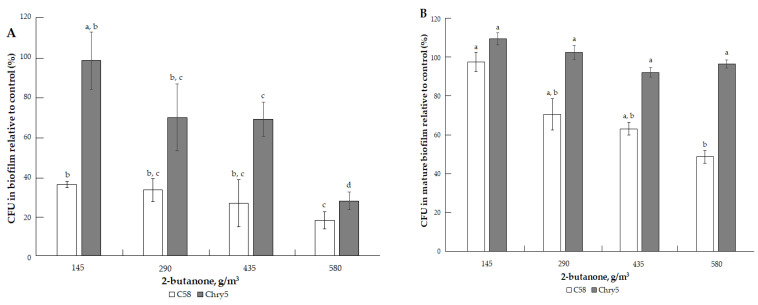
The effect of 2-butanone on the formation of biofilm of *A. tumefaciens* C58 (white column) and Chry5 (gray column) (**A**) and cell survival in mature biofilms (**B**) depending on the concentration of 2-butanone. CFU of bacteria strains in the control (without VOC): 100^a^%. The different lowercase letters above the mean values indicate significant differences (*p* ≤ 0.05; Tukey’s HSD test).

**Figure 5:**
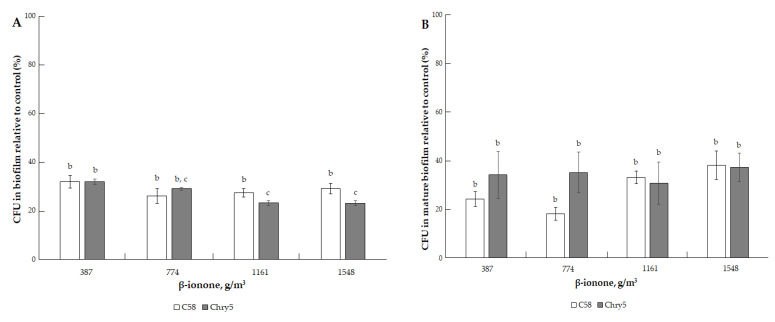
The effect of β-ionone on the formation of biofilm of *A. tumefaciens* C58 (white column) and Chry5 (gray column) (**A**) and cell survival in mature biofilms (**B**) depending on the concentration of β-ionone. CFU of bacteria strains in the control (without VOC): 100^a^%. The different lowercase letters above the mean values indicate significant differences (*p* ≤ 0.05; Tukey’s HSD test).

**Figure 6:**
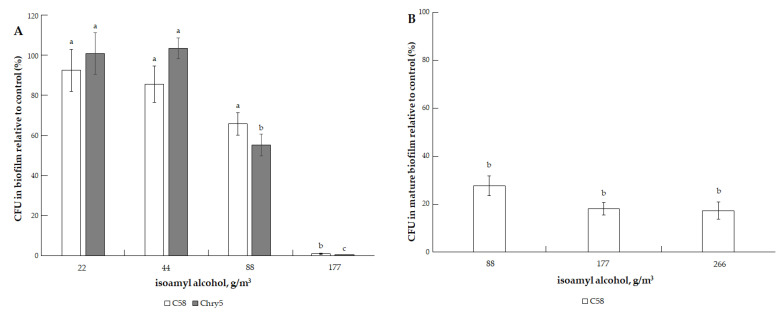

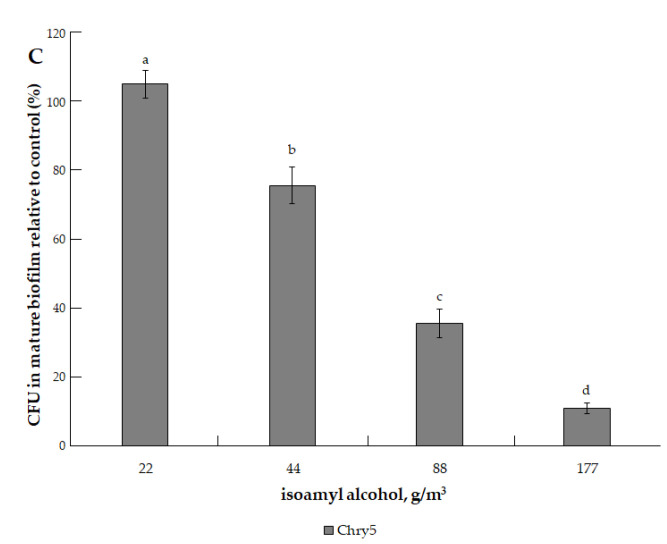
The effect of isoamyl alcohol on the formation of biofilm of *A tumefaciens* C58 (white column) and Chry5 (gray column) (**A**) and cell survival in mature biofilms of the C58 strain (**B**) and Chry5 strain (**C**) depending on the concentration of isoamyl alcohol. CFU of bacteria strains in the control (without VOC): 100^a^%. The different lowercase letters above the mean values indicate significant differences (*p* ≤ 0.05; Tukey’s HSD test).

**Figure 7:**
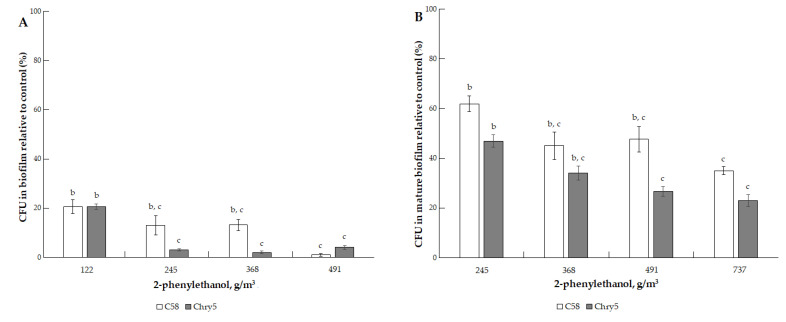
The effect of 2-phenylethanol on the formation of biofilm of *A. tumefaciens* C58 (white column) and Chry5 (gray column) (**A**) and cell survival in mature biofilms (**B**) depending on the concentration of 2-phenylethanol. CFU of bacteria strains in the control (without VOC): 100^a^%. The different lowercase letters above the mean values indicate significant differences (*p* ≤ 0.05; Tukey’s HSD test).

**Figure 8:**
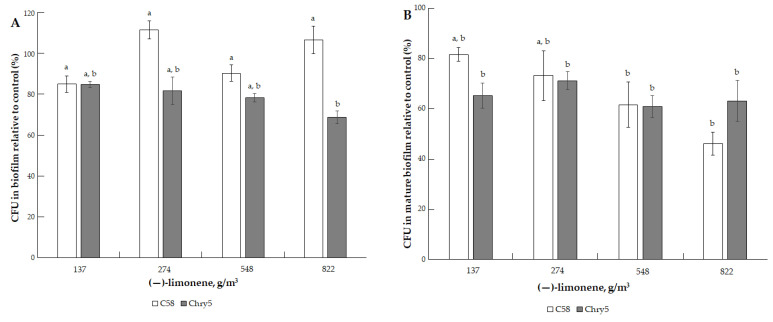
The effect of (−)-limonene on the formation of biofilm of *A. tumefaciens* C58 (white column) and Chry5 (gray column) (**A**) and cell survival in mature biofilms (**B**) depending on the concentration of (−)-limonene. CFU of bacteria strains in the control (without VOC): 100^a^%. The different lowercase letters above the mean values indicate significant differences (*p* ≤ 0.05; Tukey’s HSD test).

**Figure 9:**
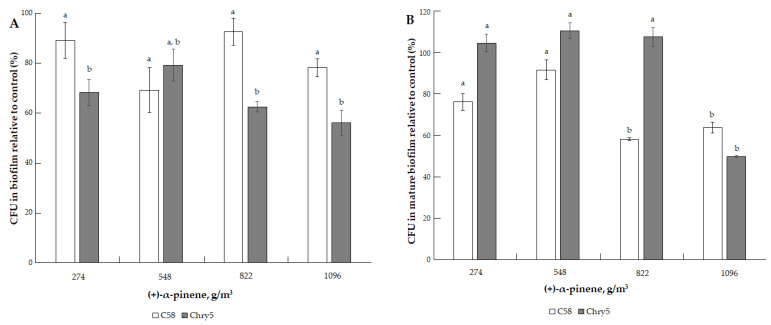
The effect of (+)-α-pinene on the formation of biofilm of *A. tumefaciens* C58 (white column) and Chry5 (gray column) (**A**) and cell survival in mature biofilms (**B**) depending on the concentration of (+)-α-pinene. CFU of bacteria strains in the control (without VOC): 100^a^%. The different lowercase letters above the mean values indicate significant differences (*p* ≤ 0.05; Tukey’s HSD test).

The results of studying the effects of the three types of VOCs (ketones, alcohols, terpenes) on the formation of colony biofilms and mature biofilms (already formed biofilms) of two strains of *A. tumefaciens* of different origins are presented in Figure 2, Figure 3, Figure 4, Figure 5, Figure 6, Figure 7, Figure 8 and Figure 9. A convenient methodological approach in this work was the method of colony biofilms. This method is suitable for treating biofilms with volatile substances and makes it easy to determine the number of cells in biofilms.

In the control, the average numbers of CFUs of *A. tumefaciens* C58 and Chry5 in the biofilms were 2.4 × 10^9^ and 3.6 × 10^9^ and in mature biofilms, 3.9 × 10^9^ and 5.3 × 10^9^, respectively.

The three ketones tested inhibited the formation of *A. tumefaciens* biofilms to varying degrees. 2-Octanone had the strongest inhibitory effect; it suppressed the formation of biofilms at low doses. For example, at 38.7 g/m^3^ of 2-octanone, the cell number of *A. tumefaciens* C58 and Chry5 CFU in the biofilms was about 20% of the control (without VOC) (Figure 2A). Cells in the mature biofilms formed by the *Agrobacterium* strains were more resistant to the action of 2-octanone (Figure 2B). The survival of 20% of the cells in the mature biofilms was observed at higher doses of 2-octanone: 129.04 (Chry5) and 258.09 g/m^3^ (C58). Effects of 2-octanone were weaker in both variants of experiments on the C58 strain. 2-Pentanone and 2-butanone inhibited biofilm formation at much higher doses: 173.4–693.5 and 145.15–580.6 g/m^3^, respectively (Figure 3A and Figure 4A). 2-Pentanone at 346.73 g/m^3^ completely inhibited the formation of biofilms of the Chry5 strain, and the CFU of the C58 strain in biofilm was 40% of the control (Figure 3A). The effect of the VOCs on the survival of bacterial cells in mature biofilms was much weaker in the case of the action of 2-pentanone on Chry5 (CFU of Chry5 was ~15% at 693.5 g/m^3^) and 2-butanone (no effect on C58 at doses of 145.15–580.6 g/m^3^) (CFU of Chry5 was 50% at 580.6 g/m^3^) (Figure 3B and Figure 4B).

The unsaturated ketone β-ionone reduced approximately by three times the number of cells in biofilms of both strains at doses of 387.07–1548.3 g/m^3^; this effect was almost the same when this VOC acted on the cells in biofilms during their formation and on mature biofilms. Interestingly, in both variants of experiments, we observed a plateau of the CFU values in the range of the amount of β-ionone of 387.07–1548.3 g/m^3^ (Figure 5A,B).

The effect of isoamyl alcohol on the biofilm formation appeared, starting from 88.7 g/m^3^, and at a dose of 177.43 g/m^3^, a sharp, almost to zero, decrease in the number of CFU in the biofilms of both strains was detected (Figure 6A). The cells of both strains in mature biofilms were more resistant to the action of isoamyl alcohol. CFU was ~20% for strain C58 and 10% for strain Chry5 at 177.43 g/m^3^ of isoamyl alcohol (Figure 6B,C). Under the action of 2-phenylethanol, the formation of biofilms (the amount of CFU in them) sharply decreased at doses of 122.94–491.78 g/m^3^ (Figure 7A). CFU of both strains was 20% of the control at 122.94 g/m^3^. Cells in mature biofilms formed by the *Agrobacterium* strains were more resistant to 2-phenylethanol (Figure 7B).

Terpene (−)-limonene had only a minor inhibition effect on the formation of *A. tumefaciens* biofilms and the survival (CFU) of cells in mature biofilms at doses of 137.11–822.69 g/m^3^ (Figure 8A,B). We observed the same patterns in the case of the action of another terpene, (+)-α-pinene, at doses of 274–1096.84 g/m^3^ (Figure 9A,B).

The action of ketones (2-pentanone, 2-octanone), terpenes, and 2-phenylethanol on the formation of biofilms of the C58 strain was weaker compared to their effect on the Chry5 strain. Only in the case of 2-butanone, the action of this VOC on the C58 strain biofilms’ formation and mature biofilms was stronger compared to the effect on the Chry5 strain. The effects of β-ionone and isoamyl alcohol on the colony biofilms of both strains were almost the same. The various effects of VOCs on the biofilms of the C58 and Chry5 strains are apparently explained by their different properties, which may be associated with the origin of these strains (isolation of strains from tumors of sweet cherries and chrysanthemums, respectively).

For the studied VOCs, the minimum bactericidal concentration (MBC) value was determined experimentally or calculated based on a set of experimental data. The value of MBC can be used as a criterion by which it is possible to evaluate the effectiveness of the action of VOCs on colony biofilms. The values of MBC are presented in the Supplementary Materials (Tables S1–S4).

### 3.2. The Action of VOCs on the Swimming Motility of A. tumefaciens

The ability of bacteria to migrate over the surface of the medium is important for the formation of biofilms [28]. We studied the ability of *A. tumefaciens* strains C58 and Chry5 regarding three types of motility: swarming, swimming, and twitching. These strains did not demonstrate swarming and twitching motility in our experiments (data not shown), which agrees with the results described by Merritt et al. (2007) [43]. 

**Figure 10:**
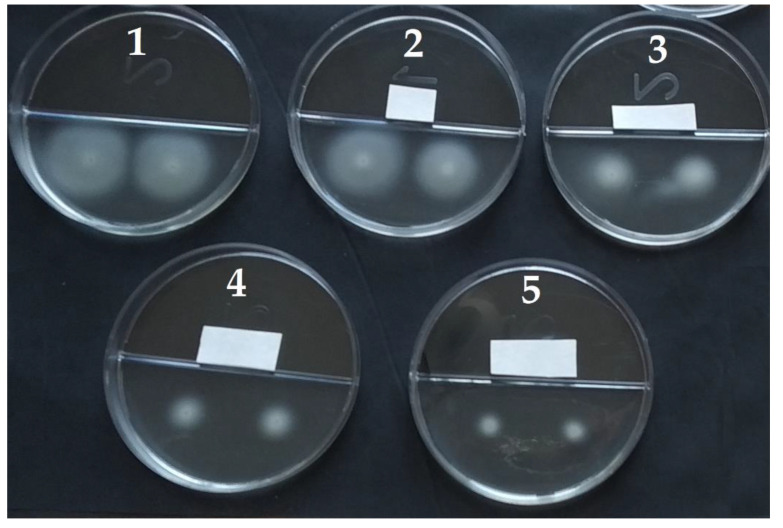
The action of 2-octanone on the swimming motility of *A. tumefaciens* C58 (1: control without VOC; 2: 12.9 g/m^3^; 3: 25.81 g/m^3^; 4: 38.71 g/m^3^; 5: 64.52 g/m^3^). Photo of a representative experiment.

Data on the effects of the tested VOCs on the swimming motility of *A. tumefaciens* strains are shown in Table 2. Swimming motility was estimated by the radius of the swimming growth zones of bacteria grown on an agarized medium. The radius of the swimming zones was measured around a grown drop of 3 µL of cell suspension (Figure 10; photo of a representative experiment). In the control (without VOCs), the average radii of the *A. tumefaciens* C58 and Chry5 motility zones were 14.3 and 14.5 mm, respectively.

It was shown that bacterial VOCs are able to suppress the swimming motility of *A. tumefaciens* C58 and Chry-5 bacteria. The ketone 2-octanone had the greatest inhibitory effect; this VOC suppressed swimming motility already at the low doses of 12.9–64.52 g/m^3^. Under the action of 2-pentanone and 2-butanone, an effect similar to that of 2-octanone at 64.52 g/m^3^ was achieved at significantly higher doses of these VOCs: at 173.37–260.05 and 435.44–580.58 g/m^3^, respectively. 

Isoamyl alcohol had an inhibitory effect on the swimming motility of the *A. tumefaciens* Chry5 strain at low doses of 22.18–88.71 g/m^3^. The same patterns were also observed under the action of 2-phenylethanol on swimming motility. β-Ionone and terpenes ((−)-limonene and (+)-α-pinene) had practically no effect on the swimming motility of both *A. tumefaciens* strains in the tested dose ranges of 48.38–387.07, 274.23–822.69, and 68.55–548.42 g/m^3^, respectively.

The action of 2-octanone and alcohols on the swimming motility of the C58 strain was weaker compared to the effect of these VOCs on the Chry5 strain. The effect of 2-pentanone and 2-butanone on the swimming motility of both strains was almost the same.

## 4. Discussion

In this work, we studied for the first time the effects of eight VOCs with a different chemical nature on biofilms formed by the phytopathogenic *A. tumefaciens* strains C58 and Chry5. These bacteria form tumors (crown galls) on plants that cause great damage to agriculture. The ability of phytopathogenic bacteria, including *Agrobacterium* species, to form biofilms on plants is one of the virulence factors in bacterial infections because these bacteria, in a biofilm state, are more resistant to plant defensive mechanisms and are proficient in plant colonization. Therefore, the study of the effects of various antibacterial compounds on biofilms is of both fundamental and applied interest. VOCs are a new source of compounds with antibiotic activity [1,3,6,8] that can inhibit the formation of biofilms by phytopathogenic bacteria.

We explored the effects on *A. tumefaciens* colony biofilms’ formation and cell survival in mature biofilms of representatives of three groups of VOCs: (1) ketones (2-octanone, 2-pentanone, 2-butanone, unsaturated ketone β-ionone), (2) alcohols (isoamyl alcohol, 2-phenylethanol), and (3) terpenes ((–)-limonene, (+)-α-pinene).

It is possible that properties of VOCs, such as volatility (vapor pressure) and lipophilicity, may affect the effectiveness of their action on biofilms; for the tested VOCs, they are presented in Table 1. The molecular weight, number of carbon atoms, boiling point, and vapor pressure indicate the compound’s volatility [44]. VOCs (studied in this and previous [24] works) are divided into two categories according to their vapor pressures (VP): 1–100 Pa = volatile compound (from a low to high value of VP: β-ionone, 2-phenylethanol, 2-undecanone, and 2-nonanone) and >100 Pa = highly volatile compound (from a low to high value of VP: 2-octanone, (–)-limonene, isoamyl alcohol, 2-heptanone, (+)-α-pinene, dimethyl disulfide, 2-pentanone, and 2-butanone) (Table 1) [42]. Lipophilicity is another important property of a compound as it influences some physiological properties, including transport through cell membranes, rate of metabolism, and interaction with receptor-binding sites. log P (log Kow) (a logarithmic form of the partition coefficient for a compound between octanol and water) serves as a measure of the relationship between the lipophilicity (fat solubility) and hydrophilicity (water solubility) of a substance. The value of log P is more than one if a substance is more soluble in fat-like solvents such as n-octanol, and less than one if it is more soluble in water [45]. All VOCs studied in this and previous [24] works are lipophilic compounds (from a low to high value of log P: 2-butanone, 2-pentanone, isoamyl alcohol, 2-phenylethanol, DMDS, 2-heptanone, 2-octanone, 2-nonanone, β-ionone, 2-undecanone, (–)-limonene, and (+)-α-pinene) (Table 1). We did not find a clear relationship between the inhibitory effect and vapor pressure (volatility) of the studied compounds nor between the inhibitory effect and value of the lipophilicity of the compounds (Table 1 and  Tables S1–S4). In addition, since we evaluated the results of the experiments after a long exposure time to VOCs (24 h), we assumed that during this time, all the VOCs evaporated. Therefore, under the conditions of the experiment, the inhibitory effect of the tested VOCs on the bacterial biofilms may not have depended directly on the rate of evaporation of these VOCs.

It was also interesting to evaluate whether the carbon chain length affects the inhibitory effect of VOC. Of the six ketones studied in this and previous [24] works, 2-nonanone and 2-octanone exhibited the most significant inhibitory effect on *A. tumefaciens* biofilm formation. In total, the degree of the severity of the effect of ketones can be represented as the following row of VOCs (from a low to high value of minimal bactericidal concentration): 2-nonanone > 2-octanone > 2-heptanone > 2-pentanone, 2-undecanone, 2-butanone > β-ionone. The effects of ketones did not fully correlate with their carbon chain length. Ketones with a chain length of nine carbon atoms (9C) (2-nonanone) and 8C (2-octanone) have the most inhibitory effect; however, 2-undecanone containing 11C has about the same inhibitory effect as the least effective 2-pentanone (5C) and 2-butanone (4C) (see the Supplementary Materials Tables S1 and S2). Probably, some other properties of this ketone influence its inhibitory action.

Since, as previously reported, ketones and other VOCs inhibit the growth of agrobacteria [16,18], it can be assumed that the main reason for the inhibition of biofilm formation and the action of VOCs on cells in mature biofilms is the suppression of bacterial growth. Indeed, the patterns of the effects of VOCs on the formation of biofilms and the survival of cells in mature biofilms correlate with the effects of VOCs on the growth of *A. tumefaciens*. An analysis of the action of VOCs studied in this work on swimming motility also shows a correlation of their effects with those on the formation of biofilms and the growth of agrobacteria. However, it is unlikely that the formation of colony biofilms depended significantly on swimming motility in our experiments, since these two processes took place under different conditions: biofilms were formed on hydrophobic filters on a solid medium, and swimming motility zones were formed on a semi-liquid nutrient medium. However, it cannot be ruled out that in natural conditions, there are possibilities for swimming motility, for example, in moist soils where agrobacteria can live.

At present, the effects of VOCs on bacterial biofilms and the mechanisms responsible for their action have been little investigated. Microarray analysis showed that VOCs 2,3-butanedione and glyoxylic acid cause global changes in *Escherichia coli* gene expression associated with motility and antibiotic resistance [46]. Some volatile organic compounds (1-butanol, 2-butanone, acetoin, ethanol, hexadecane, glyoxylic acid) slightly influenced, positively or negatively, biofilm formation in *E. coli*, *Pseudomonas aeruginosa*, *Staphylococcus* *aureus*, and *Bacillus* *subtilis*. Some of them increased or decreased the motility of these bacteria, a trait important for the formation of biofilms, colonization of bacteria, and entering plant tissues during infection [3]. We found no date on the mechanisms of action of VOCs on biofilms of *A. tumefaciens*.

Understanding the mechanisms of action of VOCs on bacterial biofilms may open new possibilities for combating biofilms and using VOCs and bacterial strains that produce them for the biocontrol of plant diseases.

## Figures and Tables

**Table 1:** Physical and chemical properties of VOCs.

VOC	M, g/mol	Vapor Pressure of VOC, mm Hg (Pa) at 25 °C	Boiling Point of VOC, °C (K) at 760 mm Hg (101.3 kPa)	Octanol: Water Partition Coefficient, log P (log K_ow_)	Amount of VOC, µmol	Concentration of VOC *, g/m^3^
**alcohols**						
2-phenylethanol (phenethyl alcohol, C_8_H_10_O)	122.16	0.0868 mm Hg (11.57 Pa) [31].	218.2 °C (491.35 K) [32].	1.36 (exp) [32].	25, 50, 100, 200, 300, 400, and 600 µmol	30.74, 61.47, 122.94, 245.89, 368.83, 491.78, and 737.67 g/m^3^
isoamyl alcohol (3-methyl-1-butanol, C_5_H_12_O)	88.15	2.37 mm Hg (315.97 Pa) [33].	131.1 °C (404.25 K) [32].	1.16 (exp) [34].	25, 50, 75, 100, 200, 300, and 400 µmol	22.18, 44.36, 66.54, 88.71, 177.43, 266.14, and 354.86 g/m^3^
**ketones**						
2-butanone (C_4_H_8_O)	72.11	90.6 mm Hg (12079 Pa) [35].	79.59 °C (352.74 K) [32].	0.26; 0.29 (exp) [34].	200, 400, 600, and 800 µmol	145.15, 290.29, 435.44, and 580.58 g/m^3^
2-pentanone (C_5_H_10_O)	86.13	35.4 mm Hg (4719.6 Pa) [33].	102.26 °C (375.41 K) [32].	0.84; 0.91 (exp) [32,34].	50, 100, 200, 300, 400, 600 and 800 µmol	43.34, 86.68, 173.37, 260.05, 346.73, 520.10, and 693.46 g/m^3^
2-heptanone ** (C_7_H_14_O)	114.19	3.85 mm Hg (513.29 Pa) [36]	151.05 °C (424.2K) [32].	1.98 [32].	25, 50, 75, 100, 200, 300, and 400 * µmol	28.73, 57.46, 86.19, 114.92, 229.84, 344.76, and 459.68 * g/m^3^
2-octanone (C_8_H_16_O)	128.22	1.35 mm Hg (179.99 Pa) [33]	172.5 °C (445.65 K) [32].	2.37 (cal) [34].	10, 20, 30, 50, 100,200, and 300 µmol	12.90, 25.81, 38.71, 64.52, 129.04, 258.09, and 387.13 g/m^3^
2-nonanone ** (C_9_H_18_O)	142.24	0.645 mm Hg (85 Pa) [34].	195.3 °C (468.45 K) [32].	3.16 (cal) [32].	5, 10, 15, 20, 25, 35, and 50 * µmol	7.16, 14.32, 21.47, 28.63, 35.79, 50.10, and 71.58 * g/m^3^
2-undecanone ** (C_11_H_22_O)	170.30	0.0975 mm Hg (13 Pa) [34].	231.5 °C (504.65 K) [32].	4.09 (cal) [32].	25, 50, 100, 200, 300, 400, and 600 * µmol	42.85, 85.70, 171.39, 342.79, 514.18, 685.57, and 1028.36 * g/m^3^
β-ionone (unsaturated ketone, C_13_H_20_O)	192.30	0.054 mm Hg (7.2 Pa) [37].	271 °C (544.15 K) [38].	3.995 (exp) [39].	25, 50, 100, 200, 400, 600, and 800 µmol	48.38, 96.77, 193.53, 387.07, 774.14, 1161.21, and 1548.28 g/m^3^
**terpenes**						
(–)-limonene (C_10_H_16_)	136.24	1.55 mm Hg (206.65 Pa) [40].	177.5 °C (450.65 K) [41].	4.57 (cal) [34].	100, 200, 400, 500, and 600 µmol	137.11, 274.23, 548.46, 685.57, and 822.69 g/m^3^
(+)-α-pinene (C_10_H_16_)	136.23	4.75 mm Hg (633.28 Pa) [31].	155.6 °C (428.75 K) [41].	4.83 (cal) [34].	50, 100, 200, 400, 600, and 800 µmol	68.55, 137.10, 274.21, 548.42, 822.63, and 1096.84 g/m^3^
**sulfur compound**						
Dimethyl disulfide ** (DMDS, C_2_H_6_S_2_)	94.19	28.7 mm Hg (3830 Pa) [34].	109.74 °C (382.89 K) [32].	1.77 (exp) [34].	50, 100, 150, 200, 300, 400, and 600 * µmol	47.40, 94.79, 142.19, 189.59, 284.38, 379.18 and 568.77 * g/m^3^

* The concentration of VOC in the Petri dish was calculated as the ratio of the amount of VOC added to the Petri dish to the headspace volume of the Petri dish (*v* = 99.4 cm^3^). ** VOCs tested in our previous work, adapted from [24]. exp = experimental; cal = calculated. The interpretation of the vapor pressures of the VOCs presented in Table 1 is as follows: 0.01–1 Pa = moderately volatile compound; 1–100 Pa = volatile compound; and >100 Pa = highly volatile compound [42].

**Table 2:** Effect of VOCs on *A. thaliana* swimming motility *.

Concentration of VOCs, g/m^3^ (μmol)	Radius of *A. tumefaciens* Swimming Zones Relative to Control (%)	
	C58	Chry5
	**2-butanone**	
145.15 (200)	(78.5 ± 2.5) ^b^	(97.7 ± 5.3) ^a^
290.29 (400)	(52.9 ± 2.5) ^c^	(60.9 ± 2.0) ^b^
435.44 (600)	(38.6 ± 4.3) ^d^	(29.9 ± 2.0) ^c^
580.58 (800)	(24.3 ± 6.6) ^e^	(25.3 ± 4.0) ^c^
	**2-pentanone**	
43.34 (50)	(82.9 ± 5.0) ^a^	(93.2 ± 2.0) ^a^
86.68 (100)	(57.1 ± 2.5) ^b^	(77.3 ± 5.2) ^b^
173.37 (200)	(42.9 ± 4.3) ^b,c^	(38.6 ± 2.0) ^c^
260.05 (300)	(32.9 ± 13.8) ^c^	(21.6 ± 7.1) ^d^
	**2-octanone**	
12.90 (10)	(85.3 ± 4.6) ^a,b^	(79.6 ± 10.4) ^b^
25.81 (20)	(81.3 ± 8.3) ^b^	(71.6 ± 3.4) ^b^
38.71 (30)	(61.3 ± 4.6) ^c^	(52.3 ± 3.9) ^c^
64.52 (50)	(41.3 ± 10.1) ^d^	(18.2 ± 7.9) ^d^
	**β-ionone**	
48.38 (25)	(98.3 ± 6.1) ^a^	(91.4 ± 4.6) ^a^
96.77 (50)	(89.5 ± 9.1) ^a^	(87.1 ± 8.9) ^a^
193.53 (100)	(91.2 ± 6.1) ^a^	(84.3 ± 6.6) ^a^
387.07 (200)	(94.7 ± 10.5) ^a^	(84.3 ± 13.8) ^a^
	**isoamyl alcohol**	
22.18 (25)	(75.7 ± 2.5) ^b^	(58.0 ± 9.0) ^b^
44.36 (50)	(78.6 ± 10.8) ^b^	(47.7 ± 3.4) ^b^
66.54 (75)	(68.6 ± 11.3) ^b^	(21.6 ± 2.0) ^c^
88.71 (100)	(68.6 ± 7.4) ^b^	(19.3 ± 5.2) ^c^
	**2-phenylethanol**	
30.74 (25)	(95.5 ± 6.8) ^a^	(69.1 ± 5.5) ^b^
61.47 (50)	(73.1 ± 2.6) ^b^	(56.0 ± 2.1) ^c^
122.94 (100)	(55.2 ± 9.3) ^c^	(33.3 ± 7.4) ^d^
245.89 (200)	(53.7 ± 7.8) ^c^	(32.1 ± 3.6) ^d^
	**(−)-limonene**	
274.23 (200)	(91.8 ± 11.4) ^a^	(94.3 ± 8.6) ^a^
548.46 (400)	(100.0 ± 7.5) ^a^	(88.6 ± 9.9) ^a^
685.57 (500)	(85.2 ± 5.7) ^a^	(87.1 ± 8.9) ^a^
822.69 (600)	(101.6 ± 20.5) ^a^	(87.1 ± 17.3) ^a^
	**(+)-α-pinene**	
68.55 (50)	(93.8 ± 9.9) ^a^	(91.2 ± 6.9) ^a^
137.10 (100)	(87.5 ± 5.7) ^a^	(98.9 ± 11.9) ^a^
274.21 (200)	(90.0 ± 6.5) ^a^	(93.4 ± 19.9) ^a^
548.42 (400)	(80.0 ± 15.2) ^a^	(96.7 ± 10.1) ^a^

* Radius of the swimming motility zones is shown as a percentage of the control (VOC was not added). The radius in the control was taken as 100^a^%. For each VOC, the different lowercase letters above the mean values indicate significant differences (*p* ≤ 0.05; Tukey’s HSD test).

## Data Availability

The data generated during the current study are available from the corresponding author on reasonable request.

## Supplementary Materials

The following supporting information can be downloaded at: https://www.mdpi.com/article/10.3390/microorganisms10081512/s1, Table S1: The minimum bactericidal concentrations of VOCs acting on *A. tumefaciens* C58 cells at biofilm formation; Table S2: The minimum bactericidal concentrations of VOCs acting on *A. tumefaciens* Chry5 cells at biofilm formation; Table S3: The minimum bactericidal concentrations of VOCs acting on *A. tumefaciens* C58 cells in mature biofilm; Table S4: The minimum bactericidal concentrations of VOCs acting on *A. tumefaciens* Chry5 cells in mature biofilm.

## References

[1] Kai M., Haustein M., Molina F., Petri A., Scholz B., Piechulla B. (2009). Bacterial Volatiles and Their Action Potential. Appl. Microbiol. Biotechnol..

[2] Effmert U., Kalderás J., Warnke R., Piechulla B. (2012). Volatile Mediated Interactions Between Bacteria and Fungi in the Soil. J. Chem. Ecol..

[3] Audrain B., Farag M.A., Ryu C.M., Ghigo J.M. (2015). Role of Bacterial Volatile Compounds in Bacterial Biology. FEMS Microbiol. Rev..

[4] Schmidt R., Cordovez V., De Boer W., Raaijmakers J., Garbeva P. (2015). Volatile Affairs in Microbial Interactions. ISME J..

[5] Tyc O., Song C., Dickschat J.S., Vos M., Garbeva P. (2017). The Ecological Role of Volatile and Soluble Secondary Metabolites Produced by Soil Bacteria. Trends Microbiol..

[6] Piechulla B., Lemfack M.C., Magnus N., Ryu C.-M., Weisskopf L., Piechulla B. (2020). Chapter 2. Bioactive Bacterial Volatiles: An Overview and Critical Comments. Bacterial Volatile Compounds as Mediators of Airborne Interactions.

[7] Veselova M.A., Plyuta V.A., Khmel I.A. (2019). Volatile Compounds of Bacterial Origin: Structure, Biosynthesis, and Biological Activity. Microbiology.

[8] Netzker T., Shepherdson E.M.F., Zambri M.P., Elliot M.A. (2020). Bacterial Volatile Compounds: Functions in Communication, Cooperation, and Competition. Annu. Rev. Microbiol..

[9] Lemfack M.C., Gohlke B.O., Toguem S.M.T., Preissner S., Piechulla B., Preissner R. (2018). MVOC 2.0: A Database of Microbial Volatiles. Nucleic Acids Res..

[10] Ryu C.M., Farag M.A., Hu C.H., Reddy M.S., Kloepper J.W., Paré P.W. (2004). Bacterial Volatiles Induce Systemic Resistance in *Arabidopsis*. Plant Physiol..

[11] Piechulla B., Degenhardt J. (2014). The Emerging Importance of Microbial Volatile Organic Compounds. Plant Cell Environ..

[12] Schulz-Bohm K., Martín-Sánchez L., Garbeva P. (2017). Microbial Volatiles: Small Molecules with an Important Role in Intra- and Inter-Kingdom Interactions. Front. Microbiol..

[13] Avalos M., van Wezel G.P., Raaijmakers J.M., Garbeva P. (2018). Healthy Scents: Microbial Volatiles as New Frontier in Antibiotic Research?. Curr. Opin. Microbiol..

[14] Fincheira P., Quiroz A. (2018). Microbial Volatiles as Plant Growth Inducers. Microbiol. Res..

[15] Weisskopf L., Schulz S., Garbeva P. (2021). Microbial Volatile Organic Compounds in Intra-Kingdom and Inter-Kingdom Interactions. Nat. Rev. Microbiol..

[16] Popova A.A., Koksharova O.A., Lipasova V.A., Zaitseva J.V., Katkova-Zhukotskaya O.A., Eremina S.I., Mironov A.S., Chernin L.S., Khmel I.A. (2014). Inhibitory and Toxic Effects of Volatiles Emitted by Strains of *Pseudomonas* and *Serratia* on Growth and Survival of Selected Microorganisms, *Caenorhabditis Elegans*, and *Drosophila Melanogaster*. Biomed Res. Int..

[17] Plyuta V.A., Chernikova A.S., Sidorova D.E., Kupriyanova E.V., Koksharova O.A., Chernin L.S., Khmel I.A. (2021). Modulation of *Arabidopsis thaliana* Growth by Volatile Substances Emitted by *Pseudomonas* and *Serratia* Strains. World J. Microbiol. Biotechnol..

[18] Sidorova D.E., Plyuta V.A., Padiy D.A., Kupriyanova E.V., Roshina N.V., Koksharova O.A., Khmel I.A. (2022). The Effect of Volatile Organic Compounds on Different Organisms: Agrobacteria, Plants and Insects. Microorganisms.

[19] Davies D.G., Parsek M.R., Pearson J.P., Iglewski B.H., Costerton J.W., Greenberg E.P. (1998). The Involvement of Cell-to-Cell Signals in the Development of a Bacterial Biofilm. Science.

[20] Costerton J.W., Stewart P.S., Greenberg E.P. (1999). Bacterial Biofilms: A Common Cause of Persistent Infections. Science.

[21] Morris C.E., Monier J.M. (2003). The Ecological Significance of Biofilm Formation by Plant-Associated Bacteria. Annu. Rev. Phytopathol..

[22] Danhorn T., Fuqua C. (2007). Biofilm Formation by Plant-Associated Bacteria. Annu. Rev. Microbiol..

[23] Rudrappa T., Biedrzycki M.L., Bais H.P. (2008). Causes and Consequences of Plant-Associated Biofilms. FEMS Microbiol. Ecol..

[24] Plyuta V., Lipasova V., Popova A., Koksharova O., Kuznetsov A., Szegedi E., Chernin L., Khmel I. (2016). Influence of Volatile Organic Compounds Emitted by *Pseudomonas* and *Serratia* Strains on *Agrobacterium tumefaciens* Biofilms. Apmis.

[25] Sciaky D., Montoya A.L., Chilton M.D. (1978). Fingerprints of *Agrobacterium* Ti Plasmids. Plasmid.

[26] Bush A.L., Pueppke S.G. (1991). Characterization of an Unusual New *Agrobacterium tumefaciens* Strain from Chrysanthemum Morifolium Ram. Appl. Environ. Microbiol..

[27] Merritt J.H., Kadouri D.E., O’Toole G.A. (2005). Growing and Analytical Statistic Biofilms. Curr. Protoc. Microbiol..

[28] Peterson S.B., Irie Y., Borlee B.R., Murakami K., Harrison J.J., Colvin K.M., Parsek M.R., Bjarnsholt T., Jensen P., Moser C., Høiby N. (2011). Chapter 15. Different Methods for Culturing Biofilms In Vitro. Biofilm Infections.

[29] Déziel E., Comeau Y., Villemur R. (2001). Initiation of Biofilm Formation by *Pseudomonas aeruginosa* 57RP Correlates with Emergence of Hyperpiliated and Highly Adherent Phenotypic Variants Deficient in Swimming, Swarming, and Twitching Motilities. J. Bacteriol..

[30] Givskov M., de Nys R., Manefield M., Gram L., Maximilien R., Eberl L., Molin S., Steinberg P.D., Kjelleberg S. (1996). Eukaryotic interference with homoserine lactone-mediated prokaryotic signalling. J. Bacteriol..

[31] Daubert T.E., Danner R.P. (1989). Physical and Thermodynamic Properties of Pure Chemicals: Data Compilation.

[32] Lide D.R. (2006). CRC Handbook of Chemistry and Physics.

[33] Riddick J.A., Bunger W.B., Sakano T.K. (1986). Techniques of Chemistry.

[34] Korpi A., Järnberg J., Pasanen A.L. (2009). Microbial volatile organic compounds. Crit. Rev. Toxicol..

[35] Alarie Y., Nielsen G.D., Andonian-Haftvan J., Abraham M.H. (1995). Physicochemical properties of nonreactive volatile organic chemicals to estimate RD50: Alternatives to animal studies. Toxicol. Appl. Pharmacol..

[36] U.S. Environmental Protection Agency DSSTox DTXSID5021916. https://comptox.epa.gov/dashboard/DTXSID5021916.

[37] Fichan I., Larroche C., Gros J.B. (1998). Water Solubility, Vapor Pressure, and Activity Coefficients of Terpenes and Terpenoids. J. Chem. Eng. Data.

[38] Sell C.S. (2006). Terpenoids. Kirk-Othmer Encyclopedia of Chemical Technology.

[39] OECD SIDS Initial Assessment Report for SIAM 20, beta-Ionone (CAS 79-77-6), April 2004, UNEP Publications. 13 July 2015. https://hpvchemicals.oecd.org/ui/handler.axd?id=27DDCF61-620E-4C8B-A7ED-606148694B0B.

[40] U.S. Environmental Protection Agency DSSTox DTXSID6047078. https://comptox.epa.gov/dashboard/DTXSID6047078.

[41] Nadais M.H., Bernardo-Gil M.G. (1993). Vapour—liquid equilibria of α-pinene + limonene at reduced pressures. Fluid Phase Equilibria.

[42] Nikunen E., Leinonen R., Kemilainen B., Kultamaa A. (2000). Environmental Properties of Chemicals.

[43] Merritt P.M., Danhorn T., Fuqua C. (2007). Motility and chemotaxis in *Agrobacterium tumefaciens* surface attachment and biofilm formation. J. Bacteriol..

[44] Sunesson A.-L. (1995). Volatile Metabolites from Microorganisms in Indoor Environments—Sampling, Analysis and Identification. Ph.D. Thesis.

[45] Saha S., Pal D., Wang Z., Wille U., Juaristi E. (2017). Chapter 14. log P. Encyclopedia of Physical Organic Chemistry.

[46] Kim K.S., Lee S., Ryu C.M. (2013). Interspecific Bacterial Sensing through Airborne Signals Modulates Locomotion and Drug Resistance. Nat. Commun..

