# Supplementary material for: Effects of Volatile Organic Compounds on Biofilms and Swimming Motility of Agrobacterium tumefaciens
Source: Microorganisms. 2022 Jul 26;10(8):1512. doi: 10.3390/microorganisms10081512 (PMC9394263; doi:10.3390/microorganisms10081512)
Supplement: Supplementary file 1 [file microorganisms-10-01512-s001.zip › microorganisms-1775030-supplementary-revision.pdf]

Table S1. The minimum bactericidal concentrations of VOCs acting on *A. tumefaciens* C58 cells at biofilms formation

VOC	MBC		R^2	Equation
	g/m³	µmol		
2-nonanone	28.63	20	—	exp
2-octanone	42.69	33.08	1	cal, $y = -7*10^{-5}*x^3 + 2,3*10^{-3}*x^2 - 29,4*10^{-3}*x + 1$
2-heptanone	114.92	100	—	exp
2-pentanone	873.27	1007.43	0.9922	cal, $y = -10^{-9}*x^3 + 3*10^{-6}*x^2 - 2,7*10^{-3} + 1$
2-butanone	n/c<br>> 580.6	n/c<br>> 800	n/c	n/c
2-undecanone	n/c<br>> 685.6	n/c<br>> 400	n/c	n/c
β-ionone	n/c<br>> 1548.3	n/c<br>> 800	n/c	n/c
isoamyl alcohol	177.43	200	0.9997	cal, $y = -10^{-5}*x^2 - 2*10^{-3}*x + 1$
2-phenylethanol	491.78	400	—	exp
DMDS	142.19	150	—	exp
(+)-α-pinene	≥1535.85*	≥1120.20*	0.5761	cal, $y = -3*10^{-9}*x^3 + 4*10^{-6}*x^2 - 1,6*10^{-3}*x + 1$
(—)-limonene	n/a<br>> 822.69	n/a<br>> 600	—	—

n/c - Experimental data set about the change of CFUs depending on the concentration of VOC in the studied range of doses is not enough to build a reliable model equation for calculating predicted minimum bactericidal concentration (MBC).

exp – the value of MBC is determined experimentally

cal – the value of MBC is calculated using a model equation that is based on an experimental data set

R^2 - the coefficient of determination measures how well a statistical model (equation) based on experimental data set predicts an outcome.

* the predicted value of MBC may not be reliable enough because $R^2 = 0.5761$ suggests that only 57.6 % of the dependent variable (y - CFU) is predicted by the independent variable (x - concentration of VOC).

n/a – VOC does not affect bacterial survival over the dose range studied in this work, so a model equation for calculating the predicted MBC cannot be built.

Table S2. The minimum bactericidal concentrations of VOCs acting on *A. tumefaciens* Chry5 cells at biofilms formation

VOC	MBC		R^2	Equation
	g/m^3	μmol		
2-nonanone	35.79	25	0,963	cal, -4,36x + 100
2-octanone	43.23	33.50	0.9584	cal, $y = -5*10^{-4}*x^2 - 3*10^{-3}*x + 1$
2-heptanone	114.92	100	—	exp
2-pentanone	327.33	377.50	1	cal, $y = 10^{-6}*x^2 - 3*10^{-3}*x + 1$
2-butanone	772.7	995.82	0.9395	cal, $y = -10^{-9}*x^3 + 6*10^{-7}*x^2 - 6*10^{-4}*x + 1$
2-undecanone	n/c<br>> 514.2	n/c<br>> 300	n/c	n/c
β-ionone	1990.91	1028.71	0.9802	cal, $y = -6*10^{-9}*x^3 + 10^{-5}*x^2 – 4,9*10^{-3}*x + 1$
isoamyl alcohol	174.48	196.68	0.948	cal, $y = -2*10^{-5}*x^2 – 1,1*10^{-3}*x + 1$
2-phenylethanol	n/c<br>> 491.78	n/c<br>> 400	n/c	n/c
DMDS	189.59	200	—	exp
(+)-α-pinene	≥ 1332.43*	≥ 971.83*	0.8591	cal, $y = -4*10^{-9}*x^3 + 5*10^{-6}*x^2 – 2,9*10^{-3}*x + 1$
(—)-limonene	1395.53	1017.78	0.9929	cal, $y = -5*10^{-9}*x^3 + 6*10^{-6}*x^2 – 1,9*10^{-3}*x + 1$

n/c - Experimental data set about the change of CFUs depending on the concentration of VOC in the studied range of dose is not enough to build a reliable model equation for calculating predicted minimum bactericidal concentration (MBC).

exp – the value of MBC is determined experimentally

cal – the value of MBC is calculated using a model equation that is based on an experimental data set

R^2 - the coefficient of determination measures how well a statistical model (equation) based on experimental data set predicts an outcome.

* the predicted value of MBC may not be reliable enough because $R^2 = 0.8591$ suggests that only 85.9 % of the dependent variable (CFU) is predicted by the independent variable (concentration of VOC).

Table S3. The minimum bactericidal concentrations of VOCs acting on *A. tumefaciens* C58 cells in mature biofilms

VOC	MBC		R^2	Equation
	g/m^3	μmol		
2-nonanone	71.58	50	—	exp
2-heptanone	344.76	300	—	exp
2-octanone	865.69	670.85	1	cal, $y = -3*10^{-8}*x^3 + 3*10^{-5}*x^2 - 8,1*10^{-3}*x + 1$
2-butanone	944.87	1301.96	0.943	cal, $y = -2*10^{-7}*x^2 - 5*10^{-4}*x + 1$
2-pentanone	1730.18	1995.98	0.9999	cal, $y = 10^{-9}*x^3 + 3*10^{-6}*x^2 - 2,5*10^{-3} + 1$
2-undecanone	n/c<br>> 1028.4	n/c<br>> 600	n/c	n/c
β-ionone	n/c<br>> 1548.3	n/c<br>> 800	n/c	n/c
isoamyl alcohol	n/c<br>> 354.86	n/c<br>> 400	n/c	n/c
2-phenylethanol	886.38	720.96	0.9843	cal, $y = -5*10^{-9}*x^3 + 6*10^{-6}*x^2 - 3,1*10^{-3} + 1$
DMDS	379.18	400	—	exp
(—)-limonene	1046.71	763.38	0.999	cal, $y = -5*10^{-9}*x^3 + 5*10^{-6}*x^2 - 2,2*10^{-3} + 1$
(+)-α-pinene	≥ 2213.4*	≥ 1614.38*	0.6386	cal, $y = -3*10^{-10}*x^3 + 6*10^{-7}*x^2 - 8*10^{-4} + 1$

n/c - Experimental data set about the change of CFUs depending on the concentration of VOC in the studied range of dose is not enough to build a reliable model equation for calculating predicted minimum bactericidal concentration (MBC).

exp – the value of MBC is determined experimentally

cal – the value of MBC is calculated using a model equation that is based on an experimental data set

R^2 - the coefficient of determination measures how well a statistical model (equation) based on experimental data set predicts an outcome.

* the predicted value of MBC may not be reliable enough because $R^2 = 0.6386$ suggests that only 63.86 % of the dependent variable (CFU) is predicted by the independent variable (concentration of VOC).

Table S4. The minimum bactericidal concentrations of VOCs acting on *A. tumefaciens* Chry5 cells in mature biofilms

VOC	MBC		R^2	Equation
	g/m^3	µmol		
2-nonanone	≥ 58.95*	≥ 41.18*	0.8041	cal, y = -3,0091x + 100
2-heptanone	459.69	400	—	exp
2-octanone	549.49	425.82	1	cal, $y = -10^{-7}*x^{3} + 7*10^{-5}*x^{2} – 1,4*10^{-2} + 1$
2-pentanone	989.78	1141.84	0.9954	cal, $y = 3*10^{-10}*x^{3} + 10^{-6}*x^{2} – 2,4*10^{-3} + 1$
2-undecanone	1578.95	921.24	0.9964	cal, $y = -5*10^{-10}*x^{3}$ + 0,0008*x2 - 0,4212*x + 100
β-ionone	2099.39	1084.76	0.9674	cal, $y = -6*10^{-9}*x^{3} + 10^{-5}*x^{2} – 4,7*10^{-3} + 1$
2-butanone	n/a<br>> 580.6	n/a<br>> 800	—	—
isoamyl alcohol	205.14	231.24	0.9176	cal, $y = 7*10^{-6}*x^{2} – 5,9*10^{-3}*x + 1$
2-phenylethanol	1478.58	1202.64	1	cal, $y = -3*10^{-9}*x^{3} + 6*10^{-6}*x^{2} – 3,7*10^{-3} + 1$
DMDS	568.77	600	—	exp
(+)-α-pinene	1308.64	954.48	0.9909	cal, $y = -5*10^{-9}*x^{3} + 4*10^{-6}*x^{2} – 3*10^{-4} + 1$
(—)-limonene	≥ 1262.6**	≥ 920.83**	0.844	cal, $y = -8*10^{-9}*x^{3} + 10^{-5}*x^{2} – 3,5*10^{-3} + 1$

n/c - Experimental data set about the change of CFUs depending on the concentration of VOC in the studied range of dose is not enough to build a reliable model equation for calculating predicted minimum bactericidal concentration (MBC).
exp – the value of MBC is determined experimentally
cal – the value of MBC is calculated using a model equation that is based on an experimental data set
R^2 - the coefficient of determination measures how well a statistical model (equation) based on experimental data set predicts an outcome.
* the predicted value of MBC may not be reliable enough because $R^2 = 0.8041$ suggests that only 80.41 % of the dependent variable (CFU) is predicted by the independent variable (concentration of VOC).
** the predicted value of MBC may not be reliable enough because $R^2 = 0.844$ suggests that only 84.4 % of the dependent variable (CFU) is predicted by the independent variable (concentration of VOC).
n/a – VOC does not affect bacterial survival over the dose range studied in this work, so a model equation for calculating the predicted MBC cannot be built.
